# *CgSCD1* Is Essential for Melanin Biosynthesis and Pathogenicity of *Colletotrichum gloeosporioides*

**DOI:** 10.3390/pathogens9020141

**Published:** 2020-02-20

**Authors:** Tan Wang, Dandan Ren, Han Guo, Xue Chen, Pinkuan Zhu, Haozhen Nie, Ling Xu

**Affiliations:** School of Life Sciences, East China Normal University, Shanghai 200241, China; 52161300002@stu.ecnu.edu.cn (T.W.); 51181300008@stu.ecnu.edu.cn (D.R.); 51171300005@stu.ecnu.edu.cn (H.G.); 52151300002@stu.ecnu.edu.cn (X.C.); pkzhu@bio.ecnu.edu.cn (P.Z.)

**Keywords:** *Colletotrichum gloeosporioides*, scytalone dehydratase, appressoria, virulence

## Abstract

*Colletotrichum gloeosporioides*, an important phytopathogenic fungus, mainly infects tropical fruits and results in serious anthracnose. Previous studies have shown that melanin biosynthesis inhibitor can inhibit the melanization of the appressoria of *Magnaporthe grisea* and *Colletotrichum* *orbiculare*, resulting in limited infection of the hosts. In this study, we identified and characterized a scytalone dehydratase gene (*CgSCD1*) from *C. gloeosporioides* which is involved in melanin synthesis. The *CgSCD1* gene deletion mutant *ΔCgscd1* was obtained using homologous recombination. The *ΔCgscd1* mutant showed no melanin accumulation on appressoria formation and vegetative hyphae. Furthermore, the virulence of *ΔCgscd1* was significantly reduced in comparison with the wild-type (WT) strain. Further investigations showed that the growth rate as well as germination and appressorium formation of *ΔCgscd1* displayed no difference compared to the wild-type and complemented transformant Cgscd1^com^ strains. Furthermore, we found that the appressorial turgor pressure in the *ΔCgscd1* mutant showed no difference compared to that in the WT and Cgscd1^com^ strains in the incipient cytorrhysis experiment. However, fewer infectious hyphae of *ΔCgscd1* were observed in the penetration experiments, suggesting that the penetration ability of nonpigmented appressoria was partially impaired. In conclusion, we identified the *CgSCD1* gene, which is involved in melanin synthesis and pathogenicity, and found that the melanization defect did not affect appressorial turgor pressure in *C. gloeosporioides*.

## 1. Introduction

*Colletotrichum gloeosporioides* is a fungal phytopathogen that causes devastation of crop and ornamental plants worldwide, resulting in substantial economic losses. During the infection process, *C. gloeosporioides* produces a series of specialized infection structures, including germ tubes, appressoria, infectious hyphae, and necrotrophic hyphae [1]. The host infection process can be roughly divided into two stages, biotrophy and necrotrophy, based on differences in the infection strategy [2]. Melanized appressoria are firstly produced at the top of the germ tubes, generated by conidia-penetrated host surfaces through a combination of mechanical forces and enzymatic degradation; the spherical biotrophic hyphae in living host cells (biotrophy stage) are then transformed and differentiated into thin, fast-growing secondary hyphae, thus causing the host tissue to be destroyed rapidly (necrotrophy stage) [1].

Most filamentous fungi have the ability to produce dark-pigmented melanin, which is used to protect organisms against destructive environmental stresses such as UV radiation, extreme temperatures, and strong oxidants [3,4,5]. As an amorphous polymer, the production of melanin can be divided into two common synthesis strategies, which are L-3,4-dihydroxyphenylalanine (L-dopa) melanin formed by the polymerization of phenolic compounds and 1,8-dihydroxynaphthalene (DHN) melanin formed by a polyketide synthase and subsequent polymerization [6].

There are plenty of related studies on the DHN melanin synthesis pathway in *Colletotrichum orbiculare*, which is a pathogen that causes anthracnose disease of cucurbit plants, particularly cantaloupe and cucumber [7]. The process of melanogenesis starts with pentaketide 1,3,6,8-tetrahydroxynaphthalene (T4HN), directly formed by polyketide synthase (transcribed by *PKS1*), and proceeds to the synthesis of scytalone by T4HN reductase (transcribed by *T4HR1*) and 1,3,8-trihydroxynaphthalene (T3HN) reductase (transcribed by *THR1*). From scytalone, the synthesis process needs to go through two dehydration steps by *SCD1* and one reduction step by *THR1*, including dehydration of scytalone to T3HN, reduction of T3HN to vermelone, and dehydration of vermelone to 1,8-DHN, which are polymerized and oxidized to obtain melanin [8]. The crucial enzymes of melanin synthesis, transcribed by *PKS1*, *T4HR1*, *THR1*, *SCD1*, and a transcription factor of melanin synthesis (*CMR1*), have been systematically studied as important biosynthetic enzymes as well as pathogenic factors of *C. orbiculare* [9,10,11,12].

The biological functions of DHN melanin in various fungi species have been explored through albino mutants which include random mutations and targeted knockout mutants, suggesting that DHN melanin plays an important role in fungal life cycles. Melanin promotes virulence by increasing resistance to hydrogen peroxide in human pathogen *Talaromyces marneffei* [13]. In the filamentous ascomycete *Sordaria macrospora*, melanin biosynthesis is related to fruiting body development and is controlled by specific regulatory genes involved in sexual differentiation [14].

Previous studies of rice blast pathogen *Magnaporthe grisea* have shown that melanized appressoria play an important role in mechanical penetration. Melanin-deficient mutants are unable to infect intact plants. The mechanical pressures caused by the increased osmotic pressure inside melanized appressoria and strong adhesives may be essential to the penetration process [15,16,17,18]. In *Colletotrichum kahawae*, the pathogen of coffee berry disease, unmelanized appressoria induced by tricyclazole show turgor pressure as low as one-quarter that of melanized appressoria, accompanied by a decrease in infection [19]. The albino mutant of *C. graminicola* is unable to penetrate intact leaves, resulting in decreased virulence. However, the accumulation of osmolytes and the generation of turgor in appressoria are independent of melanin [20]. Related studies in *Phakopsora pachyrhizi* have shown similar results, with turgor pressure accumulation in appressoria also found to be independent of melanin [21]. The causes of this phenomenon, probably due to the diversity of species and the functions of appressoria, are not completely consistent during the infection processes.

To investigate the synthesis pathway and function of melanin in the appressorial formation and penetration process in *C. gloeosporioides*, we identified and characterized the *CgSCD1* gene, which encodes for scytalone dehydratase in the melanin biosynthesis pathway. We then established a *CgSCD1* deletion mutant, *△Cgscd1*, which showed no melanization and decreased virulence. In a further study, we found that the decreased virulence of the *ΔCgscd1* mutant was due to the damaged penetration ability of appressoria but that this was not related to turgor pressure, which was unchanged. We also found that CgSCD1 on the cytoplasm of melanized appressoria, on the germ tubes, and on the germinated conidia.

## 2. Results

### 2.1. Cloning of the Scytalone Dehydratase Gene from C. gloeosporioides

**Figure 1:**
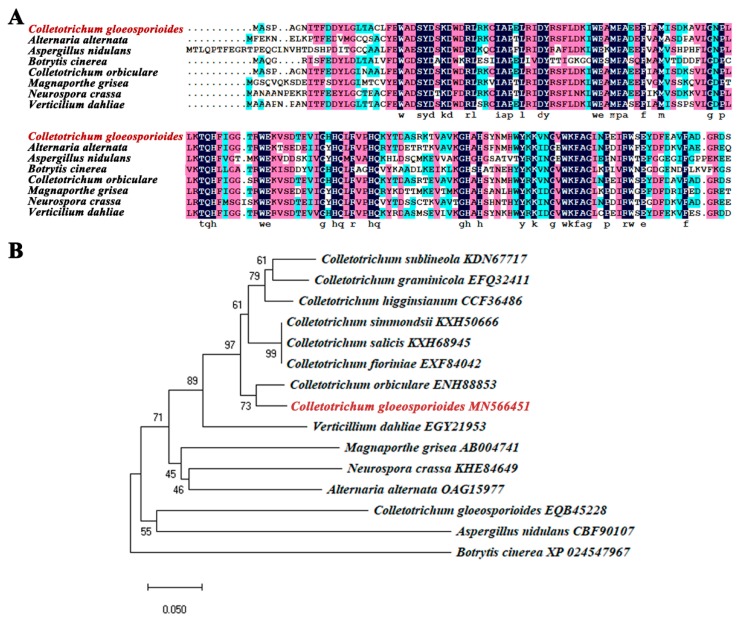
Bioinformatics analysis of CgSCD1. (**A**) Comparison of the amino acid sequence of the *Colletotrichum gloeosporioides* CgSCD1 with those of *Alternaria alternata*, *Aspergillus nidulans*, *Botrytis cinerea*, *Magnaporthe grisea*, *Neurospora crassa*, and *Verticillium dahliae*. Identical amino acids are highlighted with a black background. (**B**) Phylogenetic analysis of CgSCD1 and homologous proteins from other fungi. The protein sequence (MN566451) shown in red letters was from this experiment.

The EX2016-02 strain of wild-type *C. gloeosporioides* was isolated from the postharvest fruit of key lime (*Citrus aurantifolia*) in China [22]. Sequencing results of gDNA and cDNA PCR amplification products showed that the *CgSCD1* gene was 784 bp in length (GenBank accession number: MN539625), containing two introns and encoding a homolog of scytalone dehydratases. The results of multiple sequence alignment (Figure 1A) indicated that CgSCD1 had many conserved sites identical to the sequences of other fungal species [23,24]. A phylogenetic tree was constructed to update the new functional clades using MEGA-X software. CgSCD1 displayed high identity with proteins of *C. orbiculare* which contain a conserved nuclear transport factor 2 (NTF2-like) superfamily domain. All of the above bioinformatics analyses suggest that the predicted product of *CgSCD1* may have the same function as the fungal scytalone dehydratase reported in other species. Another homologous protein EQB45228, annotated scytalone dehydratase, was also found from the reference genome data of *C. gloeosporioides* strain Cg14. However, phylogenetic tree analysis showed that it did not aggregate with the *CgSCD1* sequences of other fungal species (Figure 1B).

### 2.2. Generation of the △Cgscd1 Mutant and Complementation

**Figure 2:**
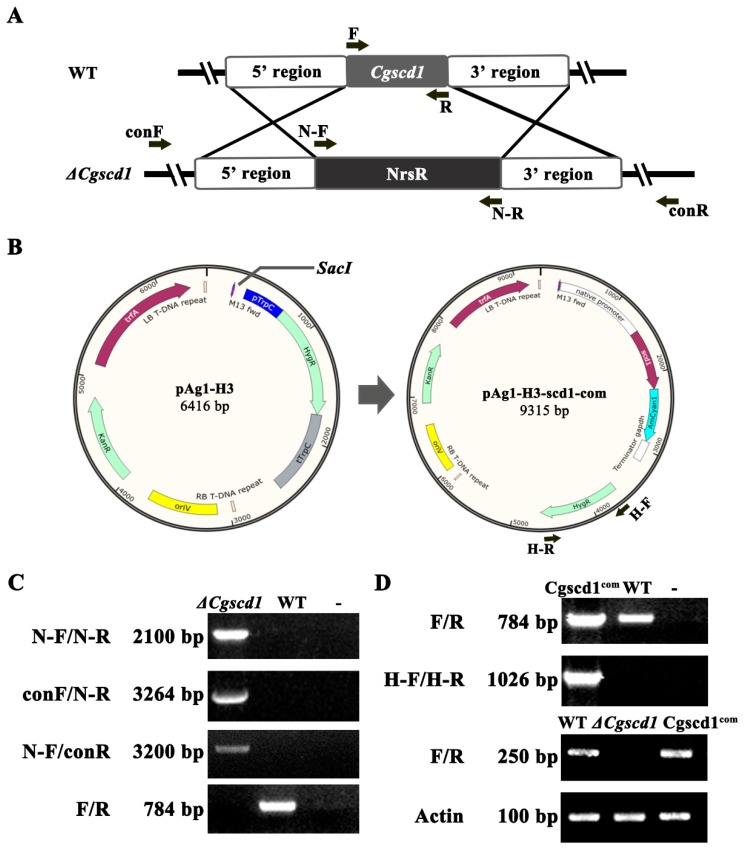
Knockout and complementary strategy of the *CgSCD1* gene, and electrophoretic identification of the *ΔCgscd1* and Cgscd1^com^ strains. (**A**) Diagram describing the homologous recombination targeted gene deletion of *CgSCD1*. (**B**) Plasmid pAg1-H3-scd1-com was used for *Agrobacterium* transformation to obtain the complementation mutant Cgscd1^com^ strain. (**C**) Confirmation of the deletion mutant *ΔCgscd1*. (**D**) Confirmation of the complementation mutant Cgscd1^com^.

To investigate the function of *CgSCD1*, a *△Cgscd1* mutant and the complementation Cgscd1^com^ strain were generated by double-crossover homologous recombination and T-DNA insertion, respectively. In the *△Cgscd1* mutant, the original *CgSCD1* sequence was replaced by NrsR cassettes, and no *CgSCD1* gene expression was detected by RT-PCR (Figure 2C,D). Fungal transformants were obtained after selection by resistance to nourseothricin. Finally, the *△Cgscd1* strain was confirmed to be an invalid mutant by sequencing and RT-PCR. The existence of a NrsR cassette was confirmed by PCR using primers N-F and N-R, with an expected product size of about 2.1 kb (Figure 2C). In addition, successful deletion in *△Cgscd1* was verified by RT-PCR, and no expression of *CgSCD1* was detected at the transcription level (Figure 2D). In the Cgscd1^com^ strain, the hygromycin resistance cassette and the *CgSCD1* gene were amplified (Figure 2C,D).

### 2.3. Characterization of *ΔCgscd1* and Cgscd1^com^

**Figure 3:**
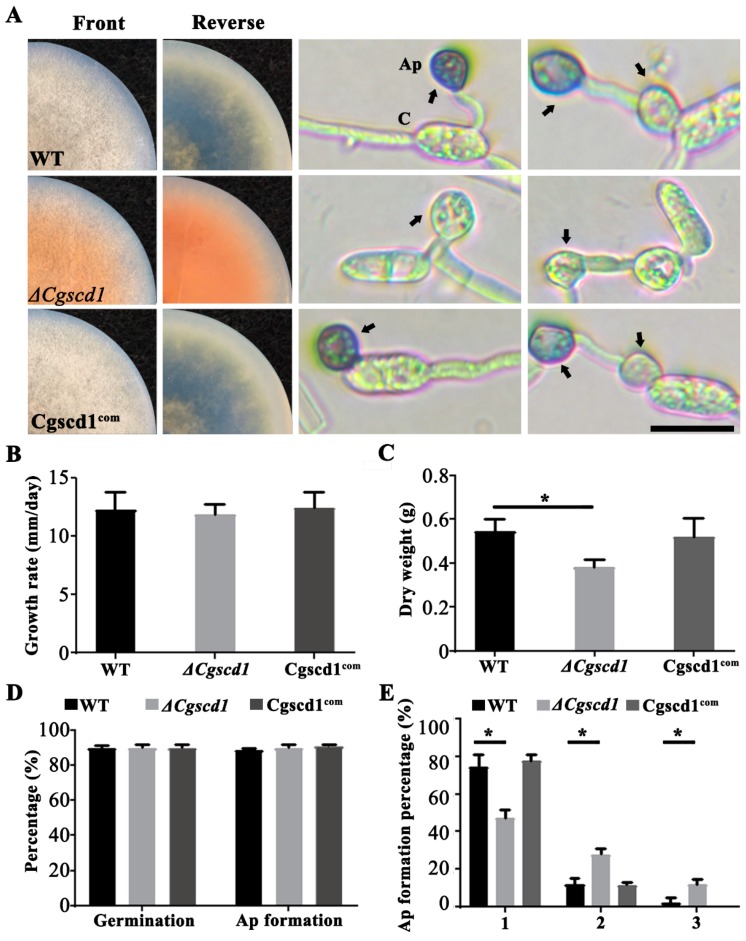
Phenotypic characteristics of the wild-type (WT), *ΔCgscd1*, and Cgscd1^com^ strains of *C. gloeosporioides*. (**A**) Colonies grown in potato dextrose agar (PDA) for 7 days, at 25 °C (images on the two columns on the left). Germinated conidia, from cultures grown for 7 days, at 25 °C (images on the two columns on the right). Arrows point to mature appressoria. Ap, appressoria; C, conidia. Scale bar (for (**A**)) = 10 µm. (**B**) Periodic (24 h) measurement of colony growth diameter. (**C**) Dry weight of mycelia grown on cellophane-covered PDA for 7 days. (**D**) Germination and appressorium formation of each strain. (**E**) Quantitative differences in the WT, *ΔCgscd1*, and Cgscd1^com^ strains in lateral germination events of appressoria (1, 2, 3 represent the number of appressoria produced by a single conidium; n = 100). For (**C**,**E**): *p* < 0.05 (*).

**Figure 4:**
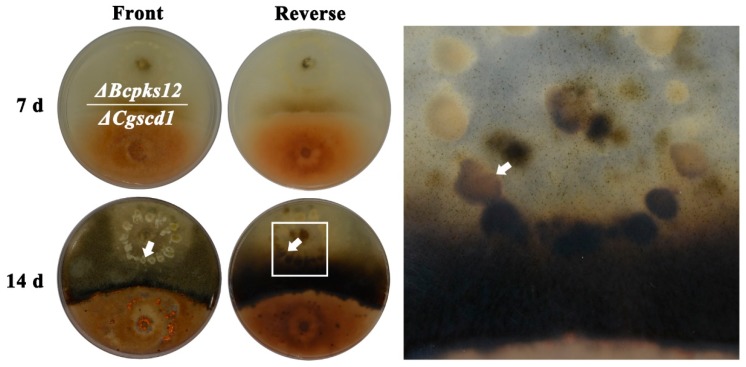
In vitro scytalone release from *ΔCgscd1*. The arrows point to melanized sclerotia, while the sclerotia far away from *ΔCgscd1* colonies are still white.

The phenotypes of the wild-type (WT), *ΔCgscd1* mutant, and Cgscd1^com^ strains were characterized by the color and growth rate of the colony, conidium germination, appressorium formation, and melanization. The *ΔCgscd1* mutant only formed colorless appressoria (Figure 3A). The Cgscd1^com^ strain showed a melanized colony and appressoria, as did the WT strain. In the *ΔCgscd1* mutant, the colonies were white, and no melanin accumulated during the initial culture. After about 7 days of incubation, the colonies and the potato dextrose agar (PDA) medium turned brown-red. This change in color may be caused by the accumulation of the scytalone, an intermediate metabolite of melanin synthesis, which was secreted into the culture medium (Figure 4) [25]. This indicates that the deletion of *CgSCD1* resulted in the albino mutant.

The growth rate of the *ΔCgscd1* strain on PDA, as determined through the calculation of colony diameter, displayed no difference compared to the WT and Cgscd1^com^ strains (Figure 3B). However, the dry weight of mycelium in the *ΔCgscd1* mutant was considerably reduced after 7 days at 25 °C compared to that in WT, and was restored in the Cgscd1^com^ strain (Figure 3C). Furthermore, in the WT, *ΔCgscd1*, and Cgscd1^com^ strains, the germination capabilities as well as appressorium formation rate were almost 90%, with no differences in germination and appressorium formation (Figure 3D). However, almost 50% of the appressoria of the *ΔCgscd1* mutant developed a secondary or tertiary appressorium. Less than 20% of the appressoria of the WT and Cgscd1^com^ strains differentiated into a secondary appressorium (Figure 3A). This suggests that the deletion of *CgSCD1* in the *ΔCgscd1* mutant affected the biomass and appressorium lateral germination.

### 2.4. In Vitro Scytalone Release from ΔCgscd1 is Able to Restore Sclerotia Melanization in ΔBcpks12

The melanin biosynthesis pathway in *Botrytis cinerea* started with the synthesis of pentaketide by *Bcpks12*, proceeded to the synthesis of scytalone, and then the scytalone went through two dehydration steps by *Bcscd1* [26]. The sclerotia of *ΔBcpks12* were light yellow from the inside core to the surface [27]. Here, we predicted that scytalone would be accumulated in the *ΔCgscd1* mutant and it may be used in the *ΔBcpks12* mutant to restore sclerotia melanization in the *ΔBcpks12* mutant. To confirm our hypothesis, a cooperation experiment was performed using *ΔCgscd1* and *ΔBcpks12*. In the test of the cooperation culture, we observed that the sclerotia formed by the *ΔBcpks12* mutant were initially white, and then turned light yellow. However, the sclerotia of the *ΔBcpks12* mutant on the side close to the *ΔCgscd1* colonies turned black after 14 days (Figure 4), indicating that scytalone from the *ΔCgscd1* mutant could be secreted into the culture medium and absorbed by *ΔBcpks12*, and could then continue to synthesize melanin in vivo to restore sclerotia melanization.

### 2.5. Analysis of *ΔCgscd1* Mutant Virulence

**Figure 5:**
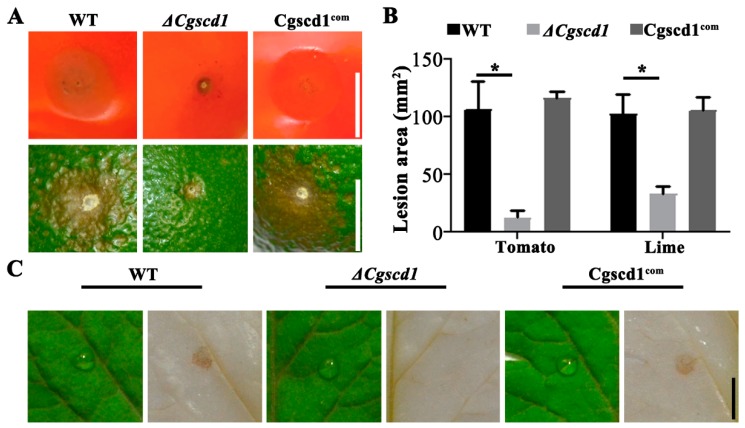
Plant infection and the virulence analysis of *ΔCgscd1* mutant. (**A**) Inoculation of tomato and lime fruits with wounds. (**B**) Analysis of the lesion area (mm^2^). (**C**) The conidial suspension was inoculated on the surface of tomato leaves without injury. The *ΔCgscd1* mutant showed almost no accumulation of hydrogen peroxide at 36 hours post-inoculation (hpi). Scale bars (for (**A**,**C**)) = 5 mm. For (**B**): *p* < 0.05 (*).

To investigate whether the *CgSCD1* gene is involved in pathogenicity, virulence analysis was performed on tomato and lime fruits, as well as tomato leaf. We found that the *ΔCgscd1* mutant failed to cause obvious sunken lesions on the fruit surface compared with the WT and Cgscd1^com^ strains (Figure 5A). Additionally, the size of the lesion area for the *ΔCgscd1* mutant was significantly reduced compared with the WT and Cgscd1^com^ strains (Figure 5B). This indicates that the deletion of *CgSCD1* affected the virulence of the *ΔCgscd1* mutant. To further study the infection ability of the *ΔCgscd1* mutant, 3,3’-diaminobenzidine (DAB) staining was used to test hydrogen peroxide accumulation on the leaf surface during infection.

The *ΔCgscd1* mutant showed almost no accumulation of hydrogen peroxide at 36 h post-inoculation (hpi), indicating that the early infection process was also limited (Figure 5C). The reason for this could be that the albino appressoria of the *ΔCgscd1* mutant may not have penetrated the host surface to form infectious hyphae, since the inoculation was performed on leaves without injury.

### 2.6. Loss of Partial Appressorium Function due to Deletion of the *CgSCD1* Gene

It has been reported that the appressoria of rice blast fungus (*M. grisea*) use glycerol to generate pressure, which ruptures plant cuticles. Melanin functions as an impermeable barrier to osmolytes, which allows appressoria to accumulate high turgor pressure [18]. Since the *ΔCgscd1* mutant had albino appressoria and showed reduced virulence, we predicted that the reduced virulence in the *ΔCgscd1* mutant was caused by a loss of appressoria function.

**Figure 6:**
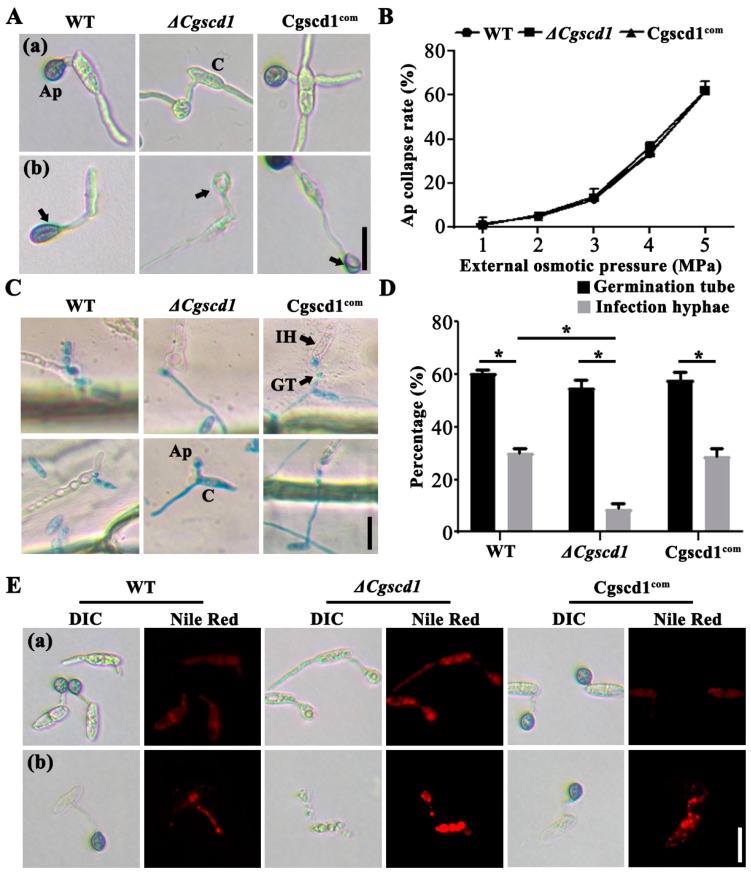
Effect of *CgSCD1* on appressorium function. (**A**) Appressoria treated with 5 MPa (osmotic pressure) PEG6000 solution and formed a longitudinal cavity. The arrows point to the longitudinal cavity. Treatment with (a) water (control); (b) PEG6000 solution. (**B**) The appressorium collapse rates of the three test strains at various osmotic pressures. (**C**) Onion epidermal infection test. (**D**) The germination tube and infection hyphae of the WT, *ΔCgscd1*, and Cgscd1^com^ strains were observed. (**E**) The effect of lysing enzymes on the integrity of appressorial cell walls. Samples were stained with Nile red. (a) Before enzymolysis. (b) After enzymolysis. Ap, appressorium; C, conidium; IH, infectious hypha; GT, germ tube. Scale bars (for (**A**,**C**,**E**)) = 10 µm. For (**D**): *p* < 0.05 (*).

Firstly, the turgor pressure of appressoria was tested in the WT, *ΔCgscd1*, and Cgscd1^com^ strains according to the method described by Howard [28]. The appressorial turgor pressure was determined by using a serial concentration of PEG6000 solution, which can lead to the collapse of appressoria when the mimic pressure is higher than the appressorial pressure. The conidia and germ tube in the WT, *ΔCgscd1*, and Cgscd1^com^ strains were shrunken, distorted, and deformed under an osmotic pressure of 5 MPa. Meanwhile, a longitudinal cavity was observed at the appressoria, indicating that the appressoria had collapsed under external osmotic pressure (Figure 6A). With the increase in osmotic pressure from 1 to 5 MPa, the trends in the appressoria collapse rates of the three tested strains were consistent, indicating that the appressorial turgor pressure was almost the same (Figure 6B). This demonstrates that deletion of the *CgSCD1* gene did not affect the appressorial internal turgor pressure of the *ΔCgscd1* mutant, although the *ΔCgscd1* mutant formed albino appressoria in which no melanin accumulated on the cell wall.

Secondly, the penetration ability in *ΔCgscd1* was tested using onion epidermal infection. In the onion epidermal infection experiment, the germ tubes and appressoria are stained by lactophenol cotton blue as they are present on the surface, while the infectious hyphae are not. The infectious hyphae can be easily identified due to them being colorless in the cotton blue staining and on the basis of their distinct morphology as vesicular structures greater in width than the germ tube (Figure 6C). The *ΔCgscd1* mutant showed no significant difference in the germination tube formation rate compared with the WT and Cgscd1^com^ strains. It was observed that the appressoria of the *ΔCgscd1* mutant successfully penetrated the onion epidermal cells. However, the formation rate of infectious hyphae in the *ΔCgscd1* mutant was extremely low compared with that in the WT strain, and could be recovered in the Cgscd1^com^ strain (Figure 6D). This indicates that deletion of the *CgSCD1* gene affected infectious hyphae formation in the *ΔCgscd1* mutant.

To test whether a defect in the production of melanin affects cell wall integration in the appressoria of *ΔCgscd1*, a Nile red staining assay was performed. Nile red is generally used to localize and quantitate lipids, particularly lipid droplets within cells. As shown in Figure 6E, the appressoria in the *ΔCgscd1* mutant were stained, but the appressoria of the WT and Cgscd1^com^ strains were not. This suggests that in comparison to WT appressoria, the *ΔCgscd1* appressoria were changed in such a way that it was easier for Nile red to penetrate the cell walls and stain the lipids inside. Treatment with lysing enzyme solution was subsequently used to test cell wall sensitivity. Following 2 h of treatment, the conidia, germ tube, and appressoria of the *ΔCgscd1* mutant could hardly be observed by light microscopy. The outlines of these structures were considerably fuzzy, and the lipid droplets were stained red and were brighter than those of the WT and Cgscd1^com^ strains (Figure 6E). After 8 h of continuous culture, the cell walls of these structures were completely digested and destroyed by lysing enzymes, leaving only spherical protoplasts. However, most of the melanized appressoria of the WT and Cgscd1^com^ strains retained their shape (Figure 6E). Taken together, the results show that albino appressoria had disintegrated and were more sensitive to lysing enzymes, suggesting that melanin has a protective effect in appressoria.

### 2.7. Localization of the CgSCD1 Protein

**Figure 7:**
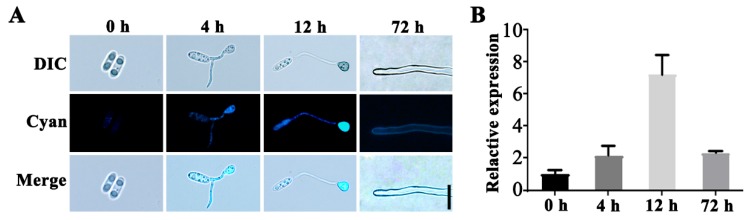
Subcellular localization of *CgSCD1* at different stages. (**A**) Cyan fluorescent protein (CFP) fluorescence was detected during the stages of germination, appressorium formation, and vegetative hyphae. Scale bar = 10 µm. (**B**) qPCR analysis of the *CgSCD1* transcript levels. Bars indicate standard deviations of three biological repeats.

To further investigate the function of the *CgSCD1* gene, the subcellular localization of the CgSCD1 protein and the transcription pattern of the *CgSCD1* gene in *C. gloeosporioides* were studied. The levels of cyan fluorescent protein (CFP) fluorescence were assayed at different developmental stages (0, 4, 12, and 72 h) for different structures, including conidia, conidia with initial appressorium formation, conidia with melanized appressorium formation, and vegetative hyphae. The signal was hardly observed in ungerminated conidia, although it increased along with the germination of conidia and eventually reached a maximum level at the melanized appressoria formation stage. The CFP fluorescence levels were observed to correspond with the levels of *CgSCD1* gene transcription as determined by qPCR. The signals of CFP fluorescence coincide with the melanization in appressoria at 12 h. Since the signals appeared to be evenly distributed throughout the appressoria, germ tube, and conidia, the CgSCD1 protein likely localized to the cytoplasm in the early germination stage. In vegetative hyphae, we found that the signal could be observed on hyphae cell walls (Figure 7A).

## 3. Discussion

The correlation between melanin and pathogenicity in different fungal species differs due to pathogen diversity. Melanogenic genes are dispensable in virulence in necrotrophic pathogen *B. cinerea* [26]. In hemibiotrophic pathogens such as *M. grisea* and *C. orbiculare*, melanized appressoria play an important role in mechanical penetration, and melanin-deficient mutants cannot infect intact plants. However, little is known about the specific mechanism and role of *SCD1* information in virulence. In this study, we firstly cloned and identified the *CgSCD1* gene in *C. gloeosporioides*.

The reference genome database of the Cg14 strain possesses two scytalone dehydratases genes, including the MN566451 (designed in this experiment) and the EQB45228. Phylogenetic tree of fungal scytalone dehydratase orthologs showed that the MN566451 clustered with SCD from *Colletotrichum* spp. However, the EQB45228 showed lower similarity in the phylogentic tree. The EQB45228 may not have functional redundancy with the MN566451 in the melanin synthesis pathway since the MN566451 deletion mutant *△Cgscd1* showed melanin synthesis deficiency in this study. It has been reported that there are two predicted scytalone dehydratases genes in *B. cinerea* and one is presumed to be a non-functional gene [26]. The EQB45228 may work somewhere else or just be a non-functional gene, which should be investigated by the deletion mutant of the EQB45228 next.

As an intermediate metabolite of the melanin biosynthesis, scytalone was accumulated and secreted into the medium due to the deletion of *CgSCD1* in the *△Cgscd1* mutant. It is not yet possible to confirm the proportion of scytalone secreted into the medium, but the scytalone remaining inside the fungi does not seem to affect most growth and development of themselves, except for a slight decrease in biomass. Similar phenotypes have been described in previous studies of *Bipolaris oryzae* [29] and *B. cinerea* [26]. Interestingly, although there was no difference in the number of appressoria formation among the WT, *△Cgscd1*, and Cgscd1^com^ strains, the proportion of secondary and tertiary appressorium of *△Cgscd1* mutant was increased, presumably due to a feedback effect caused by the unsuccessful penetration behavior of albino appressoria.

Melanin facilitates glycerol accumulation, increasing osmotic pressure in appressoria, which is essential to the penetration process in rice blast pathogen *M. grisea*. Melanin-deficient mutants of *M. grisea* are unable to infect intact plants. However, studies in *Phakopsora pachyrhizi* and *C. graminicola* showed that there was no change in turgor pressure in albino appressoria [20,21]. In this study, we tested the appressoria turgor pressure of the *△Cgscd1* mutant and found that the defect in the production of melanin has no effect on appressoria turgor pressure in *C. gloeosporioides.* It is still not clear how appressoria maintain osmotic pressure and generate high turgor pressure without melanin.

In the assays of penetrating ability, the *ΔCgscd1* mutant appressoria successfully penetrated onion epidermal cells, but the number of infectious hyphae were extremely low compared to that in the WT strain. This indicates that appressoria penetration is partially impaired in the *ΔCgscd1* mutant. The proportion of secondary and tertiary appressoria of the *△Cgscd1* mutant was increased, presumably due to a feedback effect caused by the unsuccessful penetration behavior of albino appressoria. We detected that turgor pressure was unchanged. In the Nile red staining assay, the *ΔCgscd1* mutant was more easily stained and more susceptible to lysing enzyme. Thus, the cell wall integrity may be changed, which may affect appressoria penetration in the *ΔCgscd1* mutant.

Judging from the intensity of the fluorescent signals at continuous time intervals during the early germination stage of conidia, we found that the signals in melanized appressoria were much stronger than the immature ones, indicating that CgSCD1 performs its function mainly in melanized appressoria. Interestingly, the fluorescence signals of PKS1 in *C. graminicola* were visible in immature appressoria but not in the mature appressoria with full melanization [20]. We could infer the reasons based on the melanin synthesis pathway: the PKS1 is involved in the first step of the melanin synthesis pathway, and performs its function at the earlier stage (immature appressoria), whereas *CgSCD1*, located downstream of the melanin synthesis pathway, performs its function mainly at the later stage (melanized appressoria). Of course, due to the diversity in fungal species, it may be necessary to characterize a *PKS1* mutant of *C. gloeosporioides* in order to verify our hypothesis.

## 4. Materials and Methods

### 4.1. Fungal Strains and Culture Conditions

The strains used in this study were derived from the WT strain [22] (Table 1). With the exception of strains with specific requirements, all strains were cultured on potato dextrose agar (PDA) medium at 25 °C. The 2-day-old cultured mycelia grown on a cellophane-coated PDA Petri dishes were collected with a cell scraper for DNA and RNA extraction. The liquid medium used in the test was potato dextrose broth (PDB), which was shaken at 180 rpm at 25 °C. The conidia for appressorial formation assay and host inoculation were obtained by scraping 7-day cultures from PDA Petri dishes flooded with sterilized water.

### 4.2. Nucleotide Sequencing and Sequence Analysis

Genomic DNA (gDNA) and total RNA were extracted following the method described by Dubey [30]. The complementary DNA (cDNA) was obtained using Reverse Transcription Kit (Takara, Beijing, China). The *CgSCD1* gene sequences of gDNA and cDNA were amplified by PCR amplification with the primers F and R (Table 2), respectively. Nucleotide sequencing was performed by Sangon (Sangon Biotech, Shanghai, China). The sequences were submitted to the GenBank database (accession number: MN539625, MN566451). Reference SCD1 protein sequences from other fungi were downloaded from the GenBank database. These SCD1 protein sequences, including OAG15977 in *Alternaria alternate*, CBF90107 in *Aspergillus nidulans*, XP_024547967 in *Botrytis cinerea*, AB004741 in *Magnaporthe grisea*, KHE84649 in *Neurospora crassa*, and EGY21953 in *Verticillium dahlia*, were aligned with MN566451 in *C. gloeosporioides* using DNAman 9.0 (Lynnon Biosoft, San Ramon, CA, USA). A phylogenetic tree based on this alignment was constructed using the neighbor-joining method in MEGA-X with 1000 bootstrap replicates. Conserved domains were predicted using NCBI Conserved Domain Database (https://www.ncbi.nlm.nih.gov/Structure/cdd/wrpsb.cgi).

### 4.3. Fungal Transformation

PEG-mediated protoplast transformation was used for the targeted deletion of *CgSCD1*, as described by Chung [31]. Fungal protoplasts were also prepared according to a method in that paper but with some modifications. Briefly, 2 mL conidial suspension (10^6^ conidia per mL) was added into potato dextrose broth (PDB), which was shaken at 180 rpm for 24 h at 28 °C. The hyphae were harvested by filtering through four layers of cheesecloth, and then washed three times by sterilized water. The collected hyphae were digested in 30 mL permeation buffer (1.2 M KCl) containing 0.3 g of lysing enzyme from *Trichoderma harzianum* (L1412, Sigma-Aldrich, USA) at 28 °C with gentle shaking for 4 h. The digested solution was filtered through lens cleaning papers and centrifugated at 3000× *g* for 10 min to harvest protoplasts. The protoplasts were suspended with 100 µL STC buffer (1.2 M sorbitol, 50 mM Tris-HCl, pH = 8, 50 mM CaCl_2_), mixed with 10 µg DNA fragments, and then placed on ice for 15 min. Then, 800 µL PEG (40% PEG 4000 in STC buffer) solution was added with gentle shaking at room temperature for 30 min, and then mixed with 50 mL of liquid regeneration medium (RM). RM was poured into Petri dishes using 10 mL per dish, and placed at 28 °C for 14 h of recovery. The 10 mL of PDA medium containing 100 µg/mL nourseothricin was poured into dishes for the screening of transformed mutants. After 3 days of incubation, the strains that grew on the surface of the PDA medium were transferred to new selective nourseothricin PDA Petri dishes.

### 4.4. Disruption of the *CgSCD1* Gene and Verification

To generate the knockout construct of the *CgSCD1* gene, three fragments were amplified, including upstream (1165 bp) of the *CgSCD1* gene, downstream (1164 bp) of the *CgSCD1* gene, and the nourseothricin resistance (NrsR) cassette (2.1 kb) as the selection marker. The primers for the *CgSCD1* gene fragment amplification were designed according to the reference genome data of *C. gloeosporioides* strain Cg14. The primer information is listed in Table 1. The nourseothricin resistance (NrsR) cassette (2.1 kb) was amplified using the N-F and N-R primers from plasmid pNR2 [32]. The PCR mixture included 30 ng genomic DNA, 2U DNA polymerase, and PCR master mix (10149ES03, Yeasen Biotech, Shanghai, China). PCR was carried out with the following cycling program: 3 min at 98 °C, followed by 35 cycles of 98 °C for 10 s, 60 °C for 20 s, and 72 °C for 30 s, and a final extension at 72 °C for 5 min. The amplified fragments were isolated from agarose gel. The three fragments from the first-round PCR were fused by overlapping extensions in the second round of PCR. The products (4.3 kb) from the second round of PCR were used for transformation.

The deletion of the *CgSCD1* gene was confirmed on two levels. At the genome level, the *CgSCD1* gene and NrsR cassette were amplified to verify the deletion of *CgSCD1* and the insertion of NrsR cassette, respectively. Specific primers were designed outside of the cassette region (conF and conR). The primer sets of conF and N-R were used to check for correct insertion at the 5’ region, and the primer sets of N-F and conR were used to check for correct insertion at the 3’ region. The PCR fragments, approximately 3.2 kb from the 5’ and 3’ regions, respectively, were sequenced for verification. At the RNA level, the expression of *CgSCD1* in *ΔCgscd1* was tested and compared to WT and ectopic Cgscd1^com^ by RT-PCR. The actin-like gene (Cglo_15775 from genomic dates of Cg14 strain) was used as a reference gene.

### 4.5. Complementation and Cyan Fluorescent Protein (CFP) Fusion Constructs

To confirm the phenotype of *ΔCgscd1* conferred by deletion of the *CgSCD1* gene, the complementation plasmid pAg1-H3-scd1-com was constructed based on plasmid pAg1-H3 [33]. Two fragments were inserted into the *Sac*I restriction site of pAg1-H3 to generate the pAg1-H3-scd1-com construct. One was a fragment of the native promoter, along with the full-length gDNA fragment of the *CgSCD1* gene, which was amplified using the primers pAg-comF and pAg-comR. The other was a fragment of the coding sequence of *Anemonia majano* cyan fluorescent protein (CFP) and *Cochliobolus heterostrophus* glyceraldehyde-3-phosphate dehydrogenase terminator sequence, which was amplified from plasmid pflu6 using the primers CFP-F and CFP-R. The CFP fusion was designed for the subcellular study of CgSCD1. The pAg1-H3-scd1-com construct was transformed into *A. tumefaciens* strain Agl1. The *A. tumefaciens*-mediated transformation (ATMT) procedure was based on a previously described protocol but with some modifications [34]. The complementation mutant Cgscd1^com^ was selected on hygromycin medium and verified by PCR using primers H-F and H-R for the HygR cassette, and primers F and R for the *CgSCD1* gene. The PCR cycles and temperature were as follows: 98 °C for 3 min; 35 cycles (98 °C, 10 s; 60 °C, 20 s; 72 °C, 30 s); and a final extension at 72 °C for 5 min.

### 4.6. Growth and Appressorial Formation Assay

To study the growth rate, the colony diameters of the WT, *ΔCgscd1*, and Cgscd1^com^ strains (cultured as described above) were measured once a day for 5 days. To study the sporulation, the conidium was collected and calculated from 7 days of cultured strain on PDA Petri dishes. The germination rate and appressorium formation of the WT, *ΔCgscd1*, and Cgscd1^com^ strains were performed on glass slides at 25 °C for 12 h of incubation. The conidial suspension (10^6^ conidia per mL) of each strain was prepared and placed onto glass slides (20 μL droplets per slide, three replicates). The conidial germination and appressorium development were monitored using a microscope. The conidia were considered germinating when the germination tube was longer than the conidia. Using the WT strain as a control, appressorium formation was considered positive when appressoria were completely melanized.

### 4.7. Pathogenicity Assay

The fruits of two species, including tomato VF36 and key lime, were used to test the virulence of the WT, *ΔCgscd1*, and Cgscd1^com^ strains. The fruits were stabbed using a sterile needle and wounds were inoculated with 20 µL of conidial suspension (10^6^ conidia per mL) or 20 µL sterile water as a control. Ten healthy fruits were inoculated in each treatment. The fruits were kept at 25 °C with 90% relative humidity. The lesions of tomato and key lime were observed and measured at 7 and 21 days post-inoculation (dpi). Approximately 4-week-old detached tomato (VF36) leaves were used for droplet inoculation and incubated with 40 µL conidial suspension (8 × 5 μL droplets per leaf) at 25 °C with 90% relative humidity. DAB staining was used to observe the production of H_2_O_2_ in leaves at 36 hours post-inoculation (hpi) [35]. The inoculation method used for the onion epidermis test is detailed in [36]. Quantification of lactophenol cotton blue-stained fungal infection structures (germ tube, appressoria, infectious hyphae) after the infection was performed on glass slides at 24 hpi. A total of 100 conidia were counted for each strain.

### 4.8. Detection of In Vitro Scytalone Release from *ΔCgscd1*

To test whether the scytalone was released from the *ΔCgscd1* mutant, the *ΔCgscd1* and *ΔBcpks12* mutants were separately inoculated in a symmetrical orientation about 22 mm from the edge of the dishes and cultured on PDA in Petri dishes (90 mm diameter) in darkness at 25 °C for 14 days. The color of the sclerotium was observed at the location where the hyphae were in contact.

### 4.9. Turgor Pressures Assay of Appressoria and Nile Red Stain

The conidia were inoculated on glass slides. When the conidium germinated and formed melanized appressoria, the medium was replaced with a dilution series of PEG6000 solutions, corresponding to an external turgor pressure ranging from 1 to 5 MPa [37]. The percentage of collapsed appressoria was quantified after 5 min of incubation in polyethylene glycol solution. Nile red stain solution and PBS buffer were configured as previously described, but with minor modifications [20]. Melanized appressoria on glass slides were prepared, the water was removed, and lysing enzyme solutions were added. Nile red staining was performed after 2 h of digestion. The slides were examined under a fluorescence microscope, with an excitation at 543 nm and emission at 619 nm. At least 100 conidia were counted per strain.

### 4.10. Gene Expression Analysis by qPCR

qPCR was performed using the Bio-Rad CFX96 Real-Time PCR Detection System (Bio-Rad, USA). PCR amplification was performed with 2 μL of cDNA template in a 25 μL reaction mixture containing 12.5 μL UltraSYBR Mixture (CW0957, Cwbio, Beijing, China) and 2 μL primers (Qpcr-F and Qpcr-R). PCR was carried out with the following cycling program: 95 °C for 10 min and 45 cycles of 95 °C for 30 s, 55 °C for 30 s, and 72 °C for 20 s. The expected qPCR product size was 250 bp. Three biological experiments were conducted. One representative set of results is presented as mean values of 2^−ΔΔCT^ ± SE for each treatment.

### 4.11. Subcellular Localization Analysis

The Cgscd1^com^ conidial suspensions were obtained by scraping 7-day old cultures, grown in PDA, which were flooded with sterile water. These suspensions were placed onto glass-slides and incubated at 25 °C (three replicates of 20 μL droplets per slide). Cyan fluorescence was detected at 0, 4, and 12 h using a Zeiss laser scanning microscope (ZEISS, Germany). Images were taken under 40× objective and processed with ZEN 3.0 software. Cyan fluorescence in the hyphae of Cgscd1^com^ was observed on the edge of the colony after 72 h of culture on PDA Petri dishes.

### 4.12. Statistical Analysis

The results are expressed as mean ± standard deviation (SD) and statistical analyses were carried out with SPSS software (SPSS, Chicago, USA). The statistical significance of the differences observed was analyzed by the Student’s *t*-test. Differences were considered significant at *p* < 0.05 (*).

## Figures and Tables

**Table 1:** List of fungal and bacterial strains and plasmids used in this study.

Title	Description
	Strains
WT	Wild-type strain EX2016-02
*ΔCgscd1*	A knockout mutant of *CgSCD1* gene, NrsR
Cgscd1^com^	Ectopic complementation mutant from *ΔCgscd1* NrsR,HygR
Agl1	The strain used for *Agrobacterium*-mediated transformation
	Plasmids
pNR2	Contains nourseothricin resistance sequence
pflu6	Contains cyan fluorescent protein sequence
pAg1-H3	*Agrobacterium*-mediated transformation
pAg1-H3-scd1-com	Contains *CgSCD1* for complementation along with the CFP sequence

**Table 2:** List of oligonucleotides used in this study.

Oligonucleotides	Sequence 5′→3′
1F	GTCCAGATTTTCACTTCATCACG
2R	GCCCGAATCGGGAATGCGGCTCTAGTATGTCTACTTTTCGAAATTACT
3F	TGATTACTAACAGATATCAAGCTTAATATGTAATCTGGGCACGGAA
4R	TTCTCTCATCTTATTCCTTGTT
F	ATGGCGTCCCCTGCTGGCAACA
R	TTAGTGAGCCACCGCAGACTT
conF	GATCGTCACGAGGCACTCTGGG
conR	GCTCAAGACAGTGAGAAGGAAG
N-F	CTAGAGCCGCATTCCCGATTCGGGC
N-R	AAGCTTGATATCTGTTAGTAATCA
H-F	ATGAAAAAGCCTGAACTCACCG
H-R	TTCCTTTGCCCTCGGACGAGTG
pAg-comF	CGACGGCCAGTGAATTCGAGCTGTCCAGATTTTCACTTCATCACG
pAg-comR	GAACTTGTTGGACAGGGCCATGTGAGCCACCGCAGACTTGAG
CFP-F	CTCAAGTCTGCGGTGGCTCACATGGCCCTGTCCAACAAGTTC
CFP-R	CCCGGGCCTTGGTACCGAGCTGAGGCAGATTTTTGTGGTCGG
Qpcr-F	GAAGGTCTCCGATACGGAGGTC
Qpcr-R	CCTTGACATCGTTCTTCTCGTCC
Actin-F	AGCGGAAAGCCTCGCAGT
Actin-R	TGTCGTTACCATCTCGACCCA

## References

[1] Perfect S.E., Hughes H.B., O’Connell R.J., Green J.R. (1999). *Colletotrichum*: A model genus for studies on pathology and fungal-plant interactions. Fungal Genet. Biol..

[2] O’Connell R.J., Thon M.R., Hacquard S., Amyotte S.G., Kleemann J., Torres M.F., Damm U., Buiate E.A., Epstein L., Alkan N. (2012). Lifestyle transitions in plant pathogenic *Colletotrichum* fungi deciphered by genome and transcriptome analyses. Nat. Genet..

[3] Henson J.M., Butler M.J., Day A.W. (1999). The dark side of the mycelium: Melanins of Phytopathogenic Fungi. Annu. Rev. Phytopathol..

[4] Gao Q., Garcia-Pichel F. (2011). Microbial ultraviolet sunscreens. Nat. Rev. Microbiol..

[5] Gessler N., Egorova A., Belozerskaya T. (2014). Melanin Pigments of Fungi under Extreme Environmental Conditions (Review). Appl. Biochem. Microbiol..

[6] Eisenman H.C., Casadevall A. (2012). Synthesis and assembly of fungal melanin. Appl. Microbiol. Biotechnol..

[7] Damm U., Cannon P.F., Liu F., Barreto R.W., Guatimosim E., Crous P.W. (2013). The *Colletotrichum orbiculare* species complex: Important pathogens of field crops and weeds. Fungal Divers..

[8] Tsuji G., Sugahara T., Fujii I., Mori Y., Ebizuka Y., Shiraishi T., Kubo Y. (2003). Evidence for involvement of two naphthol reductases in the first reduction step of melanin biosynthesis pathway of *Colletotrichum lagenarium*. Mycol. Res..

[9] Takano Y., Kubo Y., Shimizu K., Mise K., Okuno T., Furusawa I. (1995). Structural analysis of *PKS1*, a polyketide synthase gene involved in melanin biosynthesis in *Colletotrichum lagenarium*. Mol. Gen. Genet..

[10] Perpetua N.S., Kubo Y., Yasuda N., Takano Y., Furusawa I. (1996). Cloning and characterization of a melanin biosynthetic *THR1* reductase gene essential for appressorial penetration of *Colletotrichum lagenarium*. Mol. Plant-Microbe Interact..

[11] Kubo Y., Takano Y., Endo N., Yasuda N., Tajima S., Furusawa I. (1996). Cloning and structural analysis of the melanin biosynthesis gene *SCD1* encoding scytalone dehydratase in *Colletotrichum lagenarium*. Appl. Environ. Microbiol..

[12] Tsuji G., Kenmochi Y., Takano Y., Sweigard J., Farrall L., Furusawa I., Horino O., Kubo Y. (2000). Novel fungal transcriptional activators, Cmr1p of *Colletotrichum lagenarium* and pig1p of *Magnaporthe grisea*, contain Cys2His2 zinc finger and Zn(II)2Cys6 binuclear cluster DNA-binding motifs and regulate transcription of melanin biosynthesis genes in a developmentally specific manner. Mol. Microbiol..

[13] Woo P., Tam E., Chong K., Cai J., Tung E., Ngan A., Lau S., Yuen K. (2010). High diversity of polyketide synthase genes and the melanin biosynthesis gene cluster in *Penicillium marneffei*. FEBS J..

[14] Engh I., Nowrousian M., Kück U. (2007). Regulation of melanin biosynthesis via the dihydroxynaphthalene pathway is dependent on sexual development in the ascomycete *Sordaria macrospora*. FEMS Microbiol. Lett..

[15] Howard R., Valent B. (1996). Breaking and entering: Host penetration by the fungal rice blast pathogen *Magnaporthe grisea*. Annu. Rev. Microbiol..

[16] Chumley F., Valent B. (1990). Genetic Analysis of Melanin-Deficient, Nonpathogenic Mutants of *Magnaporthe grisea*. Mol. Plant-Microbe Interact..

[17] Howard R., Ferrari M. (1990). Role of melanin in appressorium function. Experimental Mycology.

[18] Jong J., McCormack B., Smirnoff N., Talbot N. (1997). Glycerol generates turgor in rice blast. Nature.

[19] Chen Z., Nunes M., Silva M., Rodrigues C. (2004). Appressorium turgor pressure of *Colletotrichum kahawae* might have a role in coffee cuticle penetration. Mycologia.

[20] Ludwig N., Löhrer M., Hempel M., Mathea S., Schliebner I., Menzel M. (2014). Melanin is not required for turgor generation but enhances cell-wall rigidity in appressoria of the corn pathogen *Colletotrichum graminicola*. Mol. Plant-Microbe Interact..

[21] Chang H., Miller L., Hartman G. (2014). Melanin-independent accumulation of turgor pressure in appressoria of *Phakopsora pachyrhizi*. Phytopathology.

[22] Wang T., Xiang S., Ren D., Zhu P., Xu L. (2019). First Report of *Colletotrichum gloeosporioides* Causing Postharvest Fruit Rot on *Citrus aurantifolia* in China. Plant Dis..

[23] Jordan D., Basarab G., Steffens J., Lundqvist T., Pfrogner B., Schwartz R., Wawrzak Z. (1999). Catalytic mechanism of scytalone dehydratase from *Magnaporthe grisea*. Pestic. Sci..

[24] Basarab G., Steffens J., Wawrzak Z., Schwartz R., Lundqvist T., Jordan D. (1999). Catalytic mechanism of scytalone dehydratase: Site-directed mutagenisis, kinetic isotope effects, and alternate substrates. Biochemistry.

[25] Kubo Y., Suzuki K., Furusawa I., Yamamoto M. (1983). Scytalone as a natural intermediate of melanin biosynthesis in appressoria of *Colletotrichum lagenarium*. Exp. Mycol..

[26] Schumacher J. (2016). DHN melanin biosynthesis in the plant pathogenic fungus *Botrytis cinerea* is based on two developmentally regulated key enzyme (PKS)-encoding genes. Mol. Microbiol..

[27] Zhu P., Li Q., Zhang C., Na Y., Xu L. (2017). *Bcpks12* gene inactivation substantiates biological functions of sclerotium melanization in *Botrytis cinerea*. Physiol. Mol. Plant Pathol..

[28] Howard R., Ferrari M., Roach D., Money N. (1991). Penetration of hard substrates by a fungus employing enormous turgor pressures. Proc. Natl. Acad. Sci. USA.

[29] Kihara J., Moriwaki A., Ueno M., Tokunaga T., Arase S., Honda Y. (2004). Cloning, functional analysis and expression of a scytalone dehydratase gene (*SCD1*) involved in melanin biosynthesis of the phytopathogenic fungus *Bipolaris oryzae*. Curr. Genet..

[30] Dubey A., Barad S., Luria N., Kumar D., Espeso E., Prusky D. (2016). Cation-Stress-Responsive Transcription Factors SltA and CrzA Regulate Morphogenetic Processes and Pathogenicity of *Colletotrichum gloeosporioides*. PLoS ONE.

[31] Chung K., Shilts T., Li W., Timmer L. (2002). Engineering a genetic transformation system for *Colletotrichum acutatum*, the causal fungus of lime anthracnose and postbloom fruit drop of citrus. FEMS Microbiol. Lett..

[32] Kars I., McCalman M., Wagemakers L., Kan J. (2005). Functional analysis of *Botrytis cinerea* pectin methylesterase genes by PCR-based targeted mutagenesis: *Bcpme1* and *Bcpme2* are dispensable for virulence of strain B05.10. Mol. Plant Pathol..

[33] Zhang A., Lu P., Dahl-Roshak A., Paress P., Kennedy S., Tkacz J., An Z. (2003). Efficient disruption of a polyketide synthase gene (*pks1*) required for melanin synthesis through *Agrobacterium*-mediated transformation of *Glarea lozoyensis*. Mol. Genet. Genom..

[34] Mullins E., Chen X., Romaine P., Raina R., Geiser D., Kang S. (2001). *Agrobacterium*-Mediated Transformation of *Fusarium oxysporum*: An Efficient Tool for Insertional Mutagenesis and Gene Transfer. Phytopathology.

[35] Eloy Y., Vasconcelos I., Barreto A., Freire-Filho F., Oliveria J. (2015). H_2_O_2_ plays an important role in the lifestyle of *Colletotrichum gloeosporioides* during interaction with cowpea [*Vigna unguiculata* (L.) Walp.]. Fungal Biol..

[36] He P., Wang Y., Wang X., Zhang X., Tian C. (2017). The mitogen-activated protein kinase *CgMK1* governs appressorium formation, melanin synthesis, and plant infection of *Colletotrichum gloeosporioides*. Front. Microbiol..

[37] Money N. (1995). Turgor pressure and the mechanics of fungal penetration. Can. J. Bot..

